# Generation of pancreatic β cells for treatment of diabetes: advances and challenges

**DOI:** 10.1186/s13287-018-1099-3

**Published:** 2018-12-29

**Authors:** Hussain Md. Shahjalal, Ahmed Abdal Dayem, Kyung Min Lim, Tak-il Jeon, Ssang-Goo Cho

**Affiliations:** 1 Department of Stem Cell & Regenerative Biotechnology and IDASI (Incurable Disease Animal model & Stem cell Institute), Konkuk University, 120 Neungdong-ro, Gwangjin-gu, Seoul, 05029 South Korea; 2 Department of Biochemistry and Molecular Biology, Jahangirnagar University, Savar, Dhaka, 1342 Bangladesh

**Keywords:** Embryonic stem cells (ESC), Induced pluripotent stem cells (iPSC), Differentiation, Pancreatic β cell, Islet organoids, Transplantation, β Cell maturation

## Abstract

Human embryonic stem cells (hESC) and induced pluripotent stem cells (hiPSC) are considered attractive sources of pancreatic β cells and islet organoids. Recently, several reports presented that hESC/iPSC-derived cells enriched with specific transcription factors can form glucose-responsive insulin-secreting cells in vitro and transplantation of these cells ameliorates hyperglycemia in diabetic mice. However, the glucose-stimulated insulin-secreting capacity of these cells is lower than that of endogenous islets, suggesting the need to improve induction procedures. One of the critical problems facing in vivo maturation of hESC/iPSC-derived cells is their low survival rate after transplantation, although this rate increases when the implanted pancreatic cells are encapsulated to avoid the immune response. Several groups have also reported on the generation of hESC/iPSC-derived islet-like organoids, but development of techniques for complete islet structures with the eventual generation of vascularized constructs remains a major challenge to their application in regenerative therapies. Many issues also need to be addressed before the successful clinical application of hESC/iPSC-derived cells or islet organoids. In this review, we summarize advances in the generation of hESC/iPSC-derived pancreatic β cells or islet organoids and discuss the limitations and challenges for their successful therapeutic application in diabetes.

## Background

Diabetes mellitus is a life-threatening disease, and its prevalence is increasing worldwide. The available treatment can neither cure nor completely control the complications of this disorder, which results in substantial losses of life in almost all countries in the world. Furthermore, current life-long treatment strategies impose large social and economic burdens on a family. For the last few decades, human beings have been trying to develop a treatment strategy that can effectively control this disorder and save lives. Despite tremendous efforts, however, humans have been far from success in finding an effective treatment strategy for diabetes.

Type 1 diabetes results from an absolute deficiency of insulin due to T cell-mediated autoimmune destruction of pancreas β cells [1]. The current treatment for type 1 diabetes is solely dependent on the administration of exogenous insulin. Although this approach manages the disease, unwanted risks and long-term complications persist because of the inability to tightly maintain glucose levels within a normal physiological range. Complications include life-threatening episodes of hypoglycemia, as well as long-term complications that include micro- and macro-angiopathy leading to cardiovascular pathologies, kidney failure, and neuropathy. Thus, there is a need for new treatments that provide superior control of blood glucose to minimize these complications [2]. One existing approach to treating diabetes is transplantation of purified human cadaveric islets into the portal vein to replace the destroyed β cells of the patients. This procedure typically results in better glycemic control, can render patients insulin independent for prolonged periods of time, and improves overall quality of life [3, 4]. Although promising, because of difficulties such as the scarcity of cadaveric donors compared to the large number of diabetic patients, low yield of transplantable islets from cadaveric pancreases, and necessity for chronic immunosuppression to prevent rejection of the allograft [5, 6], an alternative source of surrogate cells is needed. Moreover, the number of functional β cells that can be extracted from a single cadaveric pancreas is often not enough to restore euglycemia in a single diabetic patient [7]. This also illustrates the need for alternative sources of β cells to treat the increasing number of diabetic patients.

**Fig. 1:**
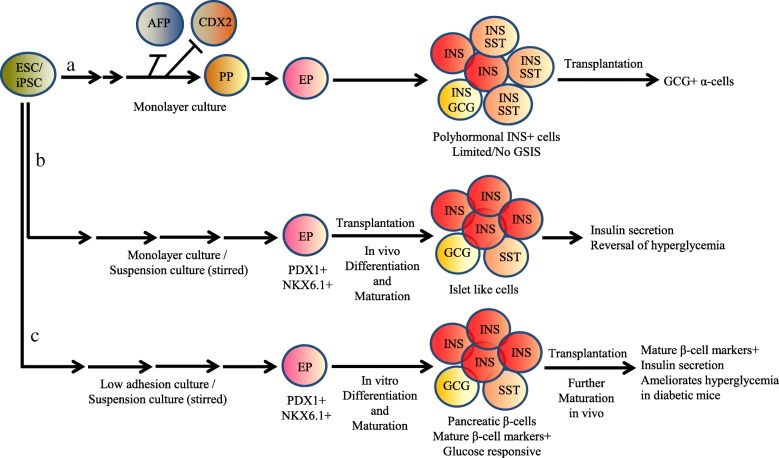
Differentiation, maturation, and function of pancreatic β cells derived from hESC/ iPSC. Insulin-positive polyhormonal cells mostly formed in many in vitro cell culture protocols which show limited or no GSIS (a). Alternatively, EP cells were formed from hESC/iPSC in a monolayer and/or rotating suspension culture, and transplantation of these cells generated islet-like ECs that exhibited GSIS and could reverse hyperglycemia (b). Recently, pancreatic β-like cells expressing mature β cell markers and exhibiting GSIS in vitro were generated in either low adhesion culture or rotating suspension culture; after transplantation, these cells underwent further maturation, secreted insulin in response to glucose, and ameliorated hyperglycemia in diabetic mice (c). GSIS, glucose-stimulated insulin secretion; AFP, hepatic progenitor cells expressing AFP; CDX2, intestinal progenitor cells expressing CDX2; PP, pancreatic progenitor; EP, endocrine precursor; INS, β-like cells expressing insulin; GCG, α cells expressing glucagon; SST, δ cells expressing somatostatin

Human pluripotent stem cells (hPSCs), including human embryonic stem cells (hESC) and induced pluripotent stem cells (hiPSC), are considered very attractive alternative sources of surrogate β cells because of their ability to differentiate into all major somatic cell lineages [8, 9]. To date, the most success in producing pancreatic β-like cells from hPSCs has come from approaches that mimic normal pancreas development. Many research groups have followed this approach, which involves exposing the cells to various growth factors and signaling molecules at specific doses and in a particular sequence to successfully differentiate the cells into pancreatic endoderm or endocrine cells (ECs) [2, 10–29]. However, in many studies, a large number of polyhormonal insulin-expressing cells have been observed in culture that resemble transient ECs seen in mid-gestation human fetal pancreases [10, 11, 15–17, 30–32]. These polyhormonal cells lack expression of key β cell transcription factors and do not secrete insulin in vitro in response to glucose challenge—the hallmark function of bona fide β cells [10, 32–34] (Fig. 1a). Concurrently, in several other studies, an alternative strategy has been adopted in which glucose-responsive insulin-secreting cells can be generated following transplantation of hESC/iPSC-derived pancreatic progenitor cells into ectopic sites in immunodeficient or type 1 diabetic mice [12, 14, 18–21, 26]. In recipient mice, the resulting cells can produce human insulin to reverse diabetes [18, 20, 21] (Fig. 1b). In recent years, optimized differentiation protocols have been successfully developed to generate glucose-responsive insulin-secreting cells in vitro from hESC/iPSC, which express mature β cell markers, and transplantation of these cells has been shown to ameliorate hyperglycemia in diabetic mice [2, 22, 23, 29] (Fig. 1c). The β-like cells generated show gene expression, ultrastructural characteristics, and glucose responsiveness both in vitro and in vivo, which closely resembling the features of β cells found in pancreatic islets [2, 22, 23]. In these multistage protocols, the final cell population has about 30–60% β-like cells, and the majority of the remaining cells are relatively uncharacterized cells that can be undifferentiated progenitors or other types of unwanted cells. Thus, improving efficiency, in terms of the percentage of differentiated cells that become β cells, remains an important challenge.

Although tremendous success has been achieved in the last few years, low survival rates of hESC/iPSC-derived pancreatic cells after transplantation into ectopic sites in recipients remain a critical problem [20, 25, 35]. Therefore, an efficient culture system that can be used to generate functional and terminally differentiated β cells, along with an effective transplantation technique, is needed for clinical application of hESC/iPSC-derived β cells for diabetes treatment. However, phase 1/2 clinical trials for the application of hESC-derived pancreatic progenitors in type 1 diabetes patients have already begun [36]. In this review, we summarize advances in the differentiation of hESC/iPSC-derived cells into pancreatic β cells and islet-like organoids and discuss the limitations and challenges for their successful generation and therapeutic application in type 1 diabetes.

## Pluripotent stem cells and their reprogramming

**Fig. 2:**
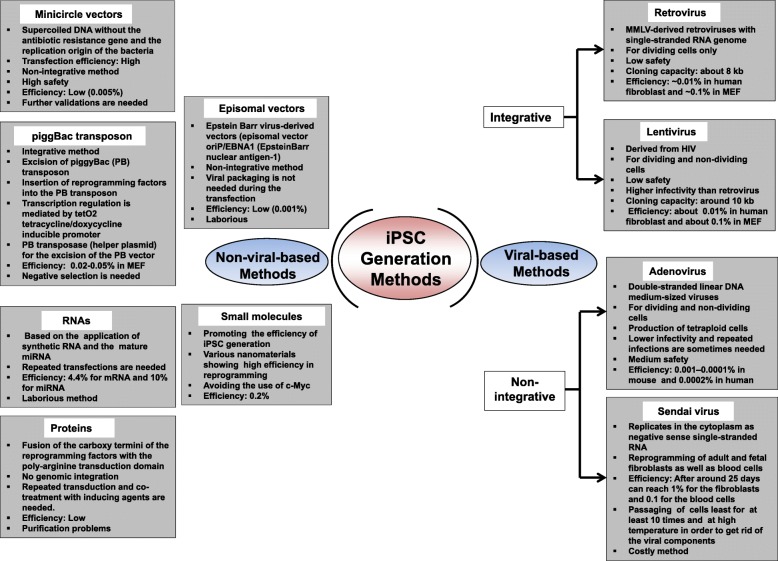
Generation of iPSCs from various somatic cells. iPSC generation carried out via viral-based and non-viral-based methods are summarized

ESCs show unlimited replicative properties and the potential to differentiate into any adult cell type [37–39]. iPSCs, established from somatic cells of mouse and human [40–42], have the same ability to expand and differentiate as ESCs. Therefore, both ESCs and iPSCs have great potential for use in cell therapies. However, the use of iPSCs has fewer ethical complications than ESCs that are derived from the inner cell mass of living embryos. iPSCs are derived from various somatic cells after exposure to a combination of transcription factors such as Oct3/4, Sox2, Klf4, and c-Myc [40, 41]. iPSC generation is carried out via viral-based and non-viral-based methods as summarized in Fig. 2. These methods are varied in their efficiencies, transduction period, genome integration, and cost [43–48]. Therefore, selection of the reprogramming method determines the further application of the produced iPSC in regenerative medicine. Generally, viral-based methods lead to genome integration and are of low safety, albeit, the high efficiency. Most iPSCs are made using retrovirus vectors, which integrate reprogramming factors into host genomes. Retrovirus vectors can spontaneously infect various cell types and insert their coding genes into host genomes using reverse transcriptase, which allows continuous transgene expression during reprogramming. Retroviral transgene expression continues until the cells become iPSCs, and then, the retroviral promoter is inactivated, possibly because of epigenetic modifications such as histone methylation [49]. This guided reprogramming and automatic silencing mechanism is considered very important for iPSC induction from somatic cells. Recently, several virus-free techniques have been developed for the production of footprint-free iPSCs; their efficient culture techniques have also been established [50–57].

## ESCs vs. iPSCs: similarities and differences

Similar to ESCs, iPSCs have a characteristic morphology, ability to generate embryoid bodies and teratomas, and unlimited proliferation capability in vitro, while they maintain their pluripotency by expressing pluripotency genes. However, several studies have revealed some differences between hESCs and hiPSCs in terms of gene expression profiles [58], epigenetic modifications such as DNA methylation [59], genetic stability [60], imprinted gene expression stability [61], differentiation potentials [62, 63], and disease modeling [64]. iPSCs have some “memory” of their somatic origin and therefore are not identical to ESCs. The memory of iPSC may affect their safety [65]. However, there is no sufficient evidence yet to determine whether iPSC memory can be fatal in cell therapies.

## Differentiation of hESC/iPSC into pancreatic β cells

Insulin-producing cells with pancreatic β cell characteristics were first successfully derived in embryoid bodies from spontaneous differentiation of hESC [66]. Since then, numerous methods to generate pancreatic endoderm or β-like cells from hESC/iPSC have been reported [2, 10–29]. These studies demonstrated the generation of insulin-positive cells, as well as glucagon- and somatostatin-positive cells (Table 1). However, the percentages of insulin-positive cells obtained in culture vary among the protocols.

**Table 1:** Generation of insulin-positive β-like cells from hESC/iPSC, their maturation, and functions in vitro and in vivo

References [Cell lines used]	Differentiation condition	Cell types induced	Percent insulin + cells	GSIS	Recipients/transplantation site	Amelioration of hyperglycemia
D’Amour et al. [10] CyT203 [hESC line]	On low-density MEFs	INS+, GCG+, SST+, PPY+, GHRL+	7.3% (3–12%)	No	n.a.	n.d.
Jiang et al. [11] H1, H9 [hESC line]	On Matrigel	INS+, GCG+, SST+ [n.t: PPY+, GHRL+]	> 15%	Yes (in vitro)	BALB/c nude mice/kidney capsule	Yes
Shim et al. [12] Miz-hES4, Miz-hES6 [hESC line]	Suspension culture	INS+, GCG+, SST+	n.d.	n.d.	BALB/c nude mice/kidney capsule	Yes
Eshpeter et al. [13] H1 [hESC line]	On Matrigel	INS+, GCG+, SST+	5.3%	Yes (in vivo)	Diabetic C57BL6 Rag −1/−1 mice/kidney capsule	No
Kroon et al. [14] CyT49, CyT203 [hESC line]	On low-density MEFs	INS+, GCG+, SST+, PPY+, GHRL+	n.d.	Yes (in vivo)	SCID-beige mice/epididymal fat pad	Yes
Chen et al. [15] HUES-2, HUES-4, HUES-8, HUES-9 [hESC line]	On low-density MEFs	INS+, GCG+, SST+, [n.t: PPY+, GHRL+]	0.8 ± 0.4%	Low (in vitro)	CD1 nude mice/kidney capsule	n.d.
Zhang et al. [16] H1, H9 [hESC line]; C1, C2, C5 [hiPSC line]	On Matrigel	INS+, SST+	25%	Yes (in vitro)	n.a.	n.d.
Kunisada et al. [17] 253G1, 201B7, 297A1, 297F1, and 297 L1 [hiPSC line]	On low-density MEFs	INS+, GCG+, SST+, GHRL+	11.8% (8.0–16.9%)	No	n.a.	n.d.
Rezania et al. [18] H1, ESI-49 [hESC line]	Matrigel/suspension culture (stirred)	INS+, GCG+, SST+, PPY+	~ 10%	Yes (in vivo)	SCID-beige and STZ-diabetic mice/kidney capsule	Yes
Schulz et al. [19] CyT49 [hESC line]	Suspension (aggregates)	INS+, GCG+, SST+, [n.t: PPY+, GHRL+]	n.d.	Yes (in vivo)	SCID-beige mice/epididymal fat pad	Yes
Rezania et al. [21] H1 [hESC line]	Matrigel/suspension culture (stirred)	INS+, GCG+, SST+, PPY+, GHRL+	~ 55–60% (post-transplant)	Yes (in vivo)	SCID-beige and STZ-diabetic mice/subcutaneous with encapsulation device	Yes
Bruin et al. [20] H1 [hESC line]	Matrigel/suspension culture (stirred)	INS+, GCG+, SST+	n.d.	Yes (in vivo)	SCID-beige and STZ-diabetic mice/kidney capsule and subcutaneous with encapsulation device	Yes
Shahjalal et al. [24] Toe, 201B7 [hiPSC]; KhES-3 [hESC line]	On Synthemax (xeno-free)	INS+, GCG+, SST+	5–8%	Yes (in vitro)	n.a.	n.d.
Rezania et al. [22] H1[hESC line]; an episomal hiPSC	Planar culture/air-liquid interface culture	INS+, GCG+, SST+	~ 50%	Yes (in vitro and in vivo)	Non-diabetic and STZ-diabetic mice/kidney capsule	Yes
Pagliuca et al. [23] HUES8 [hESC]; hiPSC-1, hiPSC-2 [hiPSC line]	Suspension culture on a stir plate	INS+, GCG+, SST+	> 30%	Yes (in vitro and in vivo)	SCID-beige and diabetic mice (NRG-Akita)/kidney capsule	Yes
Russ et al. [2] MEL1 INS^GFP/W^ [hESC line]	Low-adherence plates	INS+, GCG+, SST+	~ 60%	Yes (in vitro and in vivo)	STZ-diabetic NOD mice/kidney capsule	Yes
Agulnic et al. [25] CyT49 [hESC line]	Suspension culture	INS+, GCG+, SST+	40–50%	Yes (in vivo)	SCID-beige mice/subcutaneous with encapsulation device	n.d.
Toyoda et al. [26] KhES-3 [hESC line]; 585A1, 604B1, 692D2, 648B1 and 409B2 [hiPSC line]	On Matrigel/low adhesion plate	INS+, GCG+, SST+	n.d.	Yes (in vivo)	NOD–SCID mice/kidney subcapsule	n.d.
Millman et al. [27] ND-1 and ND-2 [ND hiPSC line]; T1D hiPSC line	On Matrigel/Spinner flasks on a stir plate	INS+, GCG+[n.t..: SST+]	24–27%	Yes (in vitro and in vivo)	ND-SCID mice and alloxan-induced diabetic mice/kidney capsule	Yes
Manzar et al. [28] T1D hiPSC line	Matrigel/3D culture	INS+, GCG+, SST+	~ 56%	Low (in vitro)	Immunodeficient STZ-diabetic mice/shoulder region	Yes
Yabe et al. [29] TkDN4-M, 253G1, 454E2 [hiPSC line]	On Matrigel/aggregate on ultra-low adhesion plate	INS+, GCG+, SST+	30–33.6%	Yes (in vitro)	STZ-diabetic NOD-SCID mice/kidney capsules	Yes

Percentages of insulin-positive cells, glucose-stimulated insulin secretion (GSIS), and in vivo functions reported from various studies are summarized

*MEFs*, mouse embryonic fibroblasts; *INS+*, insulin-positive cells; *GCG+*, glucagon-positive cells; *SST+*, somatostatin-positive cells; *PPY+*, pancreatic polypeptide-positive cells; *GHRL+*, ghrelin-positive cells; *n.t.*, not tested; *n.a.*, not applied; *n.d.*, not determined

The key stages of embryonic pancreas development include development of the definitive endoderm (DE), primitive gut tube (PG), pancreatic progenitor (PP), endocrine progenitor (EP), and hormone-expressing ECs. Based on information about embryonic pancreas development, each differentiation protocol has been designed to use various cytokines or signaling modulators at specific doses and in particular sequences to activate or inhibit key signaling pathways, including nodal/activin, Wnt, PI3K, fibroblast growth factors (FGF), bone morphogenetic protein (BMP), retinoic acid, hedgehog, protein kinase C, notch, epidermal growth factor, and transforming growth factor-β (TGF-β). Other growth factors such as insulin-like growth factors 1 and 2, hepatocyte growth factor (HGF), and glucagon-like peptide-1 (GLP-1) or exendin-4 (a peptide analog of GLP-1) have also been used to facilitate differentiation of pancreatic hormone-expressing cells [10, 11, 13, 15, 16, 24]. In addition, various classes of small molecules have been reported to be effective for differentiation of hESC/iPSC into insulin-producing cells. Nicotinamide, a poly (ADP-ribose) synthetase inhibitor, is used in some protocols to improve the yield of pancreatic ECs [11, 13, 16, 17, 24]. Further, forskolin (an activator of adenylyl cyclase) and dexamethasone (a synthetic adrenocortical steroid) have been shown to enhance cellular maturation, and these agents can be combined with other small molecules to obtain synergistic effects [17]. Thyroid hormone promotes postnatal β cell development and glucose-responsive insulin secretion in rats through the transcription factor MAFA [67]. This insight has increased the use of thyroid hormone in recent protocols to improve glucose responsiveness of hESC/iPSC-derived β cells [22, 23, 25, 27, 28, 68]. Several reports have recognized pancreatic progenitors co-expressing Pancreatic and Duodenal Homeobox 1 (PDX1) and NK6 homeobox 1 (NKX6.1) as indispensable precursors of mature pancreatic β cells [18, 21, 26]. Differentiation into pancreatic progenitors co-expressing PDX1 and NKX6.1 can be enhanced in vitro by either dissociating densely formed endodermal cells and re-plating these cells at a low density followed by exposure to a longer period of retinoid and FGF10 signaling [68] or cultures with high-density aggregates [26] or rotating a suspension culture after the addition of factors such as ALK5i (a TGF-β type I receptor kinase inhibitor II), TBP (a PKC activator), and/or LDN (a BMP inhibitor) [18, 21, 23]. The use of *epidermal growth factor* (EGF) and nicotinamide in the pancreatic progenitor specification stage can also significantly enhance pancreatic progenitor co-expressing PDX1 and NKX6.1 [69].

## Maturation of hESC/iPSC-derived β cells

The maturation of pancreatic β-like cells obtained by differentiation from hESC/iPSC in vitro remains controversial. In the early studies, either Matrigel or low-density mouse embryonic fibroblast (MEF) was used as a 2D culture platform on which hESC/iPSC were seeded [10, 11, 15–17, 30–32]. These protocols efficiently established PDX1+ progenitors by using retinoic acid in combination with inhibitors of BMP and hedgehog signaling pathways, while simultaneously adding either FGF10 or FGF7. The β-like cells generated in such monolayer culture were largely polyhormonal insulin-expressing cells (Fig. 1a). Polyhormonal cells lack expression of key β cell transcription factors and exhibit limited glucose-stimulated insulin secretion (GSIS) in vitro [10, 32–34]. Formation of non-functional polyhormonal cells is considered the limitation of these protocols. Whether the culture platform or the inappropriate combinations of growth factors in the culture media promote such cells are not clearly known. Varying degrees of in vitro GSIS from hESC/iPSC-derived insulin-positive cells have been reported by several studies, including an approximately 1.7-fold increase observed by Chen et al. [15], a 2-fold increase noted by Jiang et al. [11] and Zhang et al. [16], and apparently no GSIS reported by D’Amour et al. [10] and Kunisada et al. [17] (Fig. 1a) (Table 1). These differences and low levels of secreted insulin could be due to the generation of varying numbers of polyhormonal cells in culture. The polyhormonal cells may resemble the immature β cells observed in mid-gestation human fetal pancreases [70, 71]. The role and fate of polyhormonal cells during human fetal development are poorly understood; however, immunohistochemical characterization indicates that these cells possess an α cell transcription factor profile [72]. Several reports have described the formation of glucagon-expressing α cells in vivo following transplantation of hESC-derived polyhormonal cells [21, 33, 73] (Fig. 1a), and dynamic chromatin remodeling was reported to occur during this transition into matured cell types [73, 74]. Studies of Bruin et al. [32] revealed several key features of polyhormonal insulin-positive cells that differ from those of mature pancreatic β cells, including defects in glucose transporter expression, K_ATP_ channel function, and prohormone processing enzymes. These deficiencies must be addressed with further protocol modifications to generate hESC/iPSC-derived pancreatic β cells that show GSIS in vitro. Although several of these reports described the detection of GSIS in vitro, none of the reported cells were capable of efficiently restoring euglycemia in an in vivo diabetic animal model. To overcome this limitation, an alternative strategy to obtain glucose-responsive insulin-producing cells has been established in several studies [12, 14, 18–21, 26] (Fig. 1b). Most of these studies used Matrigel as the 2D platform for ESC/iPSC monolayer culture, followed by suspension culture with or w/o stirring using low adhesion plate. Continuous stirring promotes cell-cell and cell-matrix interactions within the culture. The resultant EP cells were then transplanted into recipient mice for further differentiation in vivo. These studies demonstrated that hESC/iPSC-derived pancreatic progenitor cells when transplanted into ectopic sites in immunodeficient or type 1 diabetes mice; they underwent further differentiation and maturation into glucose-responsive insulin-secreting cells, which could reverse diabetes in recipient mice [18, 20, 21] (Fig. 1b) (Table 1), suggesting that pancreatic precursors or immature islet-like cells obtained in vitro could mature in vivo. This also indicates that some in vivo factors are still missing in in vitro growth factor cocktails. Therefore, growth factors and signaling molecules involved in pancreas development need to be better screened to detect their potential abilities to cause hESC/iPSC to differentiate into mature pancreatic β cells in vitro.

In recent years, tremendous success has been achieved in establishing differentiation protocols that can generate glucose-responsive insulin-secreting β cells from hESC/iPSC in vitro expressing mature β cell markers, and ameliorating hyperglycemia in diabetic mice following transplantation of these cells [2, 22, 23, 29] (Fig. 1c) (Table 1). In these studies, hESCs/iPSCs were cultured either in low adhesion plate or in three-dimensional (3D) suspension culture with controlled stirring, which promote cell-cell and cell-matrix interactions resulting in the formation of cell aggregates. Sequential and time-dependent use of signaling molecules in such culture systems concurrently guides hESC/iPSC-derived cells to appropriately differentiate towards pancreatic β cells with better phenotypes in vitro. After transplantation into recipient mice, cells residing in the aggregates underwent further differentiation and maturation into mature β cells. These simplified differentiation conditions enable the efficient generation of human pancreatic and more restricted endocrine progenitor populations from pluripotent stem cells without unwanted formation of polyhormonal cells. Furthermore, the induced β cells in this protocol show both in vitro and in vivo gene expression patterns, ultrastructural characteristics, and glucose responsiveness to insulin secretion that closely resemble those of β cells from pancreatic islets [2, 22, 23]. Moreover, compared to previously reported implantations of hESC/iPSC-derived pancreatic progenitors, for which it took 3–4 months after implantation for cells to mature, recent advances in the generation of β cells in vitro substantially shorten the waiting time to therapeutic effects after implantation. In a study, Rezania et al. [22] optimized their previous differentiation protocol by adding factors such as vitamin C, protein kinase C activators, transforming growth factor-β receptor inhibitors, and thyroid hormones to generate insulin-producing cells at an induction rate of approximately 50%. Furthermore, they identified R428, a selective small-molecule inhibitor of tyrosine kinase receptor AXL, as a crucial factor for the maturation of β cells in vitro. Pagliuca et al. [23] also optimized a differentiation method to generate β cells from hESC/iPSC in vitro at an induction efficiency of > 30%. In this study, a scalable suspension-based culture system that adopted from Schulz et al. [19] was used to generate more than 10^8^ hPSCs for further differentiation. This protocol takes 4–5 weeks and involves a unique combination of sequential culture steps using factors that affect signaling in numerous pathways, including signaling by Wnt, activin, hedgehog, EGF, TGFβ, thyroid hormone, and retinoic acid, as well as γ-secretase inhibition. Later, in 2015, Russ et al. [2] showed that the use of BMP inhibitors to specify pancreatic cells promotes the precocious induction of endocrine differentiation in PDX1+ pancreatic progenitors, which ultimately results in the formation of non-functional polyhormonal cells. Therefore, in their culture system, the commonly used BMP inhibitors were omitted during pancreatic specification, which prevent precocious endocrine formation, while treatment with retinoic acid followed by combined EGF/keratinocyte growth factor (KGF) efficiently generates both PDX1+ and subsequent PDX1+/NKX6.1+ pancreatic progenitor populations, respectively. The precise temporal activation of endocrine differentiation in PDX1+/NKX6.1+ progenitors finally produces glucose-responsive β-like cells in vitro at an induction efficiency of ~ 60%. Thus, this protocol is considered to be more closely resembles key aspects of early human pancreas development and, as such, represents an improvement over previous protocols. All these observations suggest that insulin-producing cells suitable for diabetes cell therapies can be produced from hESC/iPSC in vitro. However, the formation of hESC/iPSC-induced β cells in vitro depends on multiple factors, such as the use of platforms/materials, application of suspension culture, use of a large number of growth factors and their combinations, and the timing of rotating the cultures. In these multistage protocols, although the final cell population has only about 30–60% β-like cells, the majority of the remaining cell population comprised relatively uncharacterized cells that may be undifferentiated progenitors or other types of unwanted cells. Thus, improving differentiation efficiency to generate higher percentages of β cells in vitro remains an important challenge.

A similar iPSC-derived β cell generation protocol has been reported for patients with type 1 diabetes (T1D) [27, 28]. Millman et al. [27] reported that the induced cells express β cell markers, respond to glucose both in vitro and in vivo, prevent alloxan-induced diabetes in mice, and respond to several categories of antidiabetic drugs. No major differences were observed in T1D stem cell-derived β cells compared to stem cell-induced β cells derived from non-diabetic patients. Furthermore, T1D iPSC-derived β cells responded to different forms of β cell stress in an in vitro disease model. Manzar et al. [28] generated glucose-responsive insulin-producing cells via 3D culture. In this study, T1D iPSCs were initially resistant to differentiation, but transient demethylation treatment significantly enhanced the yield of insulin-producing cells. The cells responded to high-glucose stimulation by secreting insulin in vitro. The shape, size, and number of their granules were identical to those found in cadaveric β cells. When these insulin-producing cells were transplanted into immunodeficient mice that had developed streptozotocin (STZ)-induced diabetes, hyperglycemia decreased dramatically, so that the mice become normoglycemic. Thus, T1D iPSC-derived β cells are a suitable candidate for use as an autologous cell source for the treatment of diabetes. However, a more efficient culture system is required to generate functional and terminally differentiated β cells for future research and clinical applications.

## Islet organoid generation for diabetes treatment

Pancreatic islets are composed of ECs, including insulin-producing β cells, glucagon-producing α cells, somatostatin-producing δ cells, pancreatic peptide-producing (PP) cells, and ghrelin-producing ε cells [75–77]. After functional maturation, these ECs in the islets help to regulate blood glucose levels. Reciprocal interactions among ECs in the islets are critical for regulation of insulin secretion in response to glucose [78–80]. Thus, pancreatic islet structures offer an effective means of physiologically regulating insulin secretion in patients with diabetes mellitus.

**Fig. 3:**
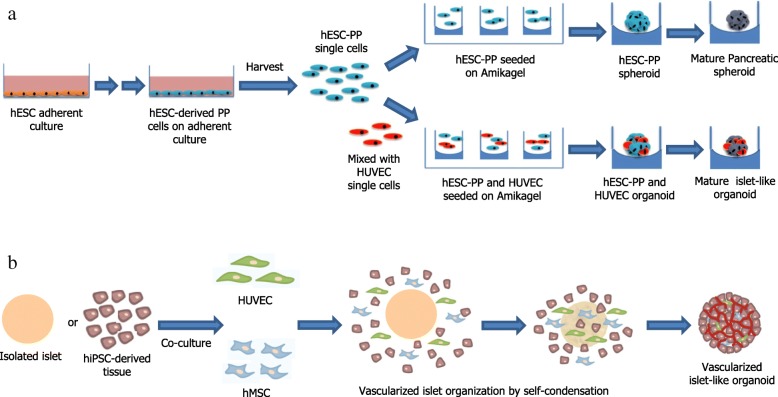
Schematic of fabrication processes for islet-like organoids, adopted and modified from Candiello et al. [82] and Takahashi et al. [87]. **a** Generation of hESC-derived islet spheroids and islet-like organoids on Amikagel hydrogel platform. Pre-differentiated hESC-derived pancreatic progenitor cells (hESC-PPs) on 2D Matrigel were harvested and then seeded onto the Amikagel hydrogel platform to either form homogenous islet spheroids or be combined with endothelial cells (HUVEC) to form endothelialized heterogeneous islet-like organoids. Several other scaffold-based strategies have also been applied to generate hESC-derived islet-like organoids such as collagen-Matrigel scaffolds. **b** Generation of vascularized islet-like organoids in self-condensation culture. In this process, isolated adult mouse/human islets or hiPSC-derived pancreatic tissues were co-cultured with endothelial cells (HUVECs) and human mesenchymal stem cells (hMSCs). In the beginning, the cells were scattered throughout the culture well, and then, they began moving towards the center of the well to form condensed tissue. Each condensed tissue contained pancreatic islets with endothelial cells surrounding them

Organoids are a group of primary cells, ESCs, or iPSCs grown in vitro that owe their self-renewal capacities and ability to differentiate into 3D structures that assume a similar organization and functionality as an organ. The generation of islet organoids containing mature β cells with full functionality is yet to be demonstrated. However, generating such functional organoids would be valuable in performing pathology studies of diabetes development, treatment, and drug screening [81, 82]. In the last decade, several research groups have reported on the generation of hESC/iPSC-derived islet-like clusters/aggregates, as well as islet-like organoids [81–87]. In the early studies, only the feasibility of generating islet-like clusters or aggregates from hESC/iPSC was studied. However, in recent years, considerable success has been achieved in generating islet-like organoids (Fig. 3). Islet-like organoids developed from hPSC by Kim et al. [86] showed glucose responsiveness in vitro, as well as in vivo. In that study, ECs expressing pancreatic endocrine hormones were first generated from hESC and iPSC using a step-wise protocol, and EC clusters (ECCs) were then formed spontaneously in 1 day from the dissociated ECs in an optimized 3D culture. The sizes of the hESC-derived ECCs were approximately 50–150 μm in diameter, which is similar to the sizes of human pancreatic islets. The ECCs comprised several pancreatic EC types, except for α cells, and thus showed that hESC-derived ECCs are, to some extent, analogous to human pancreatic islets in terms of size and cell composition. Levels of β cell-associated gene transcription and GSIS were found to be higher in ECCs than in ECs. In addition, intracellular Ca^2+^ influx oscillated in ECCs during glucose stimulation, and STZ-treated diabetic mice transplanted with ECCs became normoglycemic within 3 days after transplantation and survived for approximately 2 months. This study, therefore, supported the idea that functional islet-like organoids can be generated from hPSCs, which could serve as an alternative source of therapeutic cells for the treatment of diabetes. Another study by Wang et al. [81] demonstrated the development of islet organoids from hESC in 3D biomimetic scaffolds using several growth factors, which promote pancreatic EC differentiation. The organoids formed in this study consisted of pancreatic α, β, δ, and PP cells, and, importantly, most insulin-secreting cells generated did not co-express glucagon, somatostatin, or PP. Mature β cell marker genes were expressed, and insulin-secretory granules, which are indications of β cell maturity, were detected in these 3D-induced cell clusters. The 3D-induced organoid cells were sensitive to glucose levels; exposing the cells to a high concentration of glucose induced a sharp increase in insulin secretion. However, these islet organoids were not transplanted into animal models to confirm their biological function. The conventional 2D culture of stem cells over several passages influence cell phenotype and function [88]. Cells are only partially polarized on a flat substrate. In contrast, 3D cell culture on a specialized matrix prevents cells from attaching to the bottom of the plate by maintaining the cells in suspension or embedding them in the matrix in which cell-cell and cell-matrix interactions are maintained, thereby promises phenotypic maintenance and self-assembly of functional tissue-like polarized structures [89–91]. 3D platforms recapitulate mechanical and biochemical stimuli as present in native tissue and thus dictate polarization [92]. However, such structures often do not entirely match multicellular organization seen in native tissue. Extracellular matrix (ECM) is a critical regulator of cellular processes which serve diverse functions such as sequestering signaling molecules and transmitting ligand-specific cues via cell receptors and is amenable to synthesis, degradation, and reassembly over time [93]. Cells readily shape and remodel their extracellular environment by enzymatically degrading and resynthesizing the ECM. In tissues, ECM not only acts as an immobilization platform for a higher order of self-assembly but also facilitates the relay of biochemical and mechanical cues in a buffered and hydrated environment [93]. ECM interactions have been shown to improve β cell proliferation [94], insulin secretion [95, 96], and islet development [96–98]. Collagen has been used widely for 3D stem cell cultures [99]. A collagen scaffold constitutes a soft and flexible fibrous network that maintains cell morphology and allows cells to freely reach out, migrate, and form 3D structures. However, collagen alone is insufficient to provide multiple cues and sophisticated geometry and composition that exist in a native extracellular matrix. In previous studies, the combination of collagen with Matrigel, a complex heterogeneous mixture of basement membrane proteins such as laminin, collagen IV, fibronectin, heparin sulfate proteoglycans, and entactin, has been used as the underlying material to reconstruct cardiac muscle and uterine tissues in vitro [100, 101]. In a study, Wang et al. [81] used a four-stage differentiation strategy to differentiate hESC into pancreatic endoderm and to mature these cells into islet organoids within collagen-Matrigel scaffolds. Their results showed that augmentation of collagen scaffolds with Matrigel creates better 3D niches for islet organoid development from hESC. Although neither Matrigel nor rat tail collagen I is a US Food and Drug Administration-approved material for clinical applications, the study only demonstrated the feasibility of generating islet organoids from hESC. However, the cell clusters can be purified by enzymatically digesting the scaffolds. Another alternative is to use a porcine decellularized ECM to construct scaffolds.

A shortage of donor islets is currently limiting the widespread implementation of islet transplantation to treat diabetes [102]. In response to these needs, recent studies have focused primarily on deriving islet β cells from hESC/iPSC as an alternative to donor islets [2, 22, 23, 27–29]. The non-endocrine components of islets also play a critical role in their function. Previously, heterogeneous pancreatic organoids have been generated by aggregating adult mouse β cells with endothelial and mesenchymal cells [103]. These organoids were found to successfully integrate with host vasculature and normalize blood glucose in diabetic mice. Systematic generation of heterogeneous organoids in vitro requires an organ-specific cell source and a 3D culture platform to induce self-organization, lineage specification, functional maturation of organ-specific cells, and integration of supporting cell populations [104, 105]. Scaffold-based strategies have primarily relied on laminin-rich Matrigel, as well as other natural or synthetic biomaterials, which typically confined cells within the 3D scaffold. Recently, islet organoids have been generated from hPSCs by aggregating these cells into homogenous 3D islet-like spheroids using 2D non-adherent culture [86] or by embedding them in a collagen-Matrigel matrix [81]. Effective bioengineered platforms that will support specific organoid production, fine control over the organoid size and cellular composition, scaling up of production, and ease of organoid recovery after culture are still needed. Hydrogels have been a favored choice of materials in tissue engineering applications due to their ability to mimic the architecture and mechanics of pliable cellular microenvironment [106, 107]. Tissue-like fluidity, facile transport of soluble nutrients, ease of fabrication, and integration with biological interfaces are some of the main advantages of a hydrogel system. In a recent study, islet organoids of a precise size and cellular heterogeneity were engineered from hESC-derived pancreatic islet cells utilizing a novel hydrogel platform, Amikagel [82] (Fig. 3). The Amikagel-based platform was shown to facilitate controlled and spontaneous, rather than forced, aggregation of hESC-derived pancreatic progenitor cells (hESC-PPs) into robust homogeneous spheroids. The formation of Amikagel-induced hESC-PP spheroids enhanced pancreatic islet-specific PDX1 and NKX6.1 gene and protein expression, while also increasing the percentage of cells co-expressing both. Amikagel also enabled co-aggregation of hESC-PP with supporting endothelial cells, resulting in self-organized multicellular pancreatic organoids that were closer to islet physiology in terms of their heterogeneity than hPSC-PP homogenous spheroids^.^. These Amikagel-induced hESC-PP spheroids and heterogeneous organoids spontaneously differentiated into mature β-like cells that show expression of the β cell-specific INS1 gene, as well as C-peptide protein, and produce insulin in response to in vitro glucose challenge. After maturation, the Amikagel-induced heterogeneous organoids also show a significantly developed extracellular matrix support system. Therefore, the Amikagel platform could be ideal for engineering multicellular 3D islet organoids from hPSCs. The purpose for inducing and maintaining 3D-aggregated organoids is to enhance tissue- or organ-specific functions by reproducing cells’ native environments. Commonly used techniques for engineering islet spheroids neither have precise control over the ultimate size of an aggregate, nor do they support cell inclusion, which is the next step needed to generate islet organoids with multicellular complexity and eventual generation of vascularized constructs. The Amikagel platform is particularly suitable in this context.

A developed extracellular matrix base is important for islet structure, cell health, and overall function, while also being a prerequisite for a developed vascular system [108]. Integration of endothelial cells would be an important initial step towards vascular development [109]. In the context of regenerative medicine, tissue survival and neovessel organization of hESC-derived cells may be dependent on endothelial inclusion and mesenchymal supplementation [110]. In 2018, a complex organoid engineering method to generate pancreatic islets was reported [87] (Fig. 3). Using this protocol, pancreatic islet-like organoids with vascular networks were formed by co-culturing either isolated adult mouse/human islet tissues or hiPSC-derived pancreatic tissues with vascular endothelial cells (HUVECs) and human mesenchymal stem cells (hMSCs). Pancreatic islet-like organoids were generated by self-condensation after seeding cells onto the Matrigel bed. Transplantation of these vascularized islet-like organoids into the kidney subcapsule of fulminant type 1 diabetic mice significantly improved the survival of the diabetic mice and effectively normalized blood glucose compared to conventional islet transplantation. This approach, therefore, offers a promising alternative to therapeutic islet transplantation. The functionality of islet organoids generated so far remains partial. Some of the limitations of these organoids are that they often lack cell types needed for complete islet functions and the generation of blood vessels and nerves. To apply islet organoids to the treatment of diabetes, more complete islet structures must be prepared for better function in diabetic recipients. Despite the promise of emerging islet organoid-based approaches, developing vascular networks remains a major challenge to their application in regenerative therapies.

## ESC/iPSC-derived β cells for diabetes treatment: limitations and challenges

**Fig. 4:**
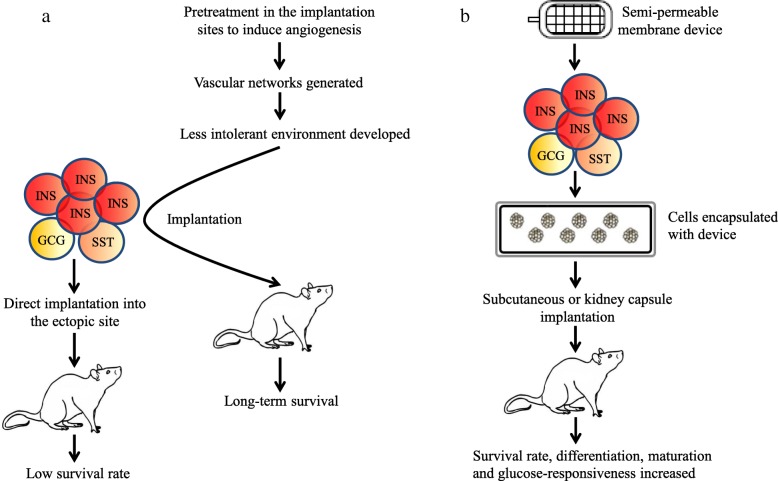
Methods for implanting hESC/iPSC-derived pancreatic cells. Two different methods have been applied. In one method, hESC/iPSC-derived pancreatic cells are implanted directly into transplantation sites or into pre-developed vascularized sites of diabetic and/or non-diabetic SCID mice (**a**). In another method, hESC/iPSC-derived pancreatic cells encapsulated in immunoprotective semipermeable devices are implanted into ectopic sites of SCID-Beige and/or diabetic mice (**b**). Oxygen, nutrients, insulin, and glucose can pass through the membranes of the devices to promote the survival, differentiation, maturation, and glucose-responsive insulin secretion of the encapsulated pancreatic cells following implantation into the host mice. In addition, vasculogenesis occurs around the devices, supporting secretion of insulin from the differentiated β cells in response to changes in glucose concentrations. In contrast, immune cells or molecules such as antibodies and complements cannot pass through the membranes, preventing immune rejection or autoimmune responses against the cells

There are still many points to address and problems to overcome before hESC/iPSC-derived cells can be clinically applied in diabetic patients, including the following: (1) Safety issues: So far, most patient-specific iPSCs have been established with retrovirus vectors. These iPSCs have numerous transgene integrations in their genomes, and these integrations may cause leaky expression that can interrupt the function of endogenous transcription factor networks and lead to differentiation failure. Another important problem of transgene integration is tumorigenic risk after transplantation. In particular, c-Myc, one of the reprogramming factors, is a well-known oncogene, and its reactivation can give rise to transgene-derived tumors in chimeric mice [111]. To make safe iPSCs, one important approach may be eliminating the c-Myc transgene in the reprogramming cocktail. Human and mouse iPSCs can be established from fibroblasts with only Oct3/4, Sox2, and Klf4, but both the efficiency of iPSC generation and the quality of these cells are significantly reduced [112]. Chimeric mice produced with c-Myc-free iPSCs did not show enhanced tumor formation in comparison with control mice. However, retroviral insertions in the genome itself may disturb endogenous gene structure and increase the risk of tumors [113]. To increase the safety of hiPSC-based cell therapies, it is necessary to generate hiPSCs without vector integration and continuous c-MYC expression. The generation of hiPSCs with transient expression from non-integrating vectors [52, 56, 114] may address these concerns. To date, various integration-free techniques have been reported, including transient expression of reprogramming factors using adenovirus [115] or Sendai virus vectors [116], the piggyBac system [51], episomal vectors [52, 56], a minicircle vector [53], and direct delivery of protein [50] or synthetic RNA [54]. However, their iPSC induction efficiencies are lower than those with retrovirus vectors, possibly because of low transduction efficiency and unstable expression [117]. (2) Variation in differentiation efficiencies: Differentiation propensities are reported to vary among hESC lines [118]. Depending on the cell origin or derivation procedure, some iPSC lines also demonstrate varying degrees of differentiation efficiency, resistance to differentiation, or tumorigenicity [65, 84]. Abnormalities in karyotype and variations in the techniques used to obtain or maintain iPSC lines and epigenetic differences among them have also been considered vital factors that alter differentiation potential. Epigenetic variations are more pronounced in iPSC than ESC. Thus, selection of good iPSC lines with low batch-to-batch variation in differentiation efficiency is essential to differentiating these cells into target lineages prior to use in specific cell therapies [118]. In addition, differentiated cells prepared from patient-specific iPSC can increase the success rate of cell therapies in the future. (3) Formation of polyhormonal cells: Polyhormonal insulin-expressing cells are frequently formed from hESC/iPSC-derived cells in vitro, as discussed above. Available reports suggest that once hESC/iPSC-derived cells become polyhormonal, they cannot be differentiated into mature β cells [33]. Therefore, it is necessary to find out the right combination of factors to reduce the formation of polyhormonal cells in culture, as well as to induce these cells into mature β cells if they are generated. (4) Xenogeneic contaminations and unknown effects: Although most recent differentiation protocols have been developed on feeder-free culture systems, many protocols still call for a variety of undefined animal-derived products that may have unknown effects on cell characteristics and differentiation ability. The potential consequences of transplanting human cells exposed to animal-derived products into patients could include increased risk of graft rejection, immunoreactions, microbial infectious, prions, and yet unidentified zoonoses [119–121]. To reduce the effects of xenogeneic contamination, Micallef et al. [122] used xeno-free media; however, they used MEF for passaging. In another study, Schulz et al. [19] expanded hESC in xeno-free media without feeder cells, but they used fetal bovine serum during differentiation. Later, Shahjalal et al. [24] expanded and differentiated hESC and iPSC in a synthetic scaffold under completely xeno-free conditions using recombinant and/or humanized components and successfully generated insulin-expressing cells in vitro. These studies indicate the feasibility of generating hESC/iPSC-derived pancreatic β cells under xeno-free conditions. For successful clinical applications, hESC/iPSC should be prepared, maintained, and differentiated in xeno-free culture systems. Humanized and/or recombinant factors, chemically defined supplements, and synthetic scaffolds can be applied in vitro to address this issue. (5) Lack of maturation: To date, insulin-expressing cells generated from hESC/iPSC in vitro lack the properties of mature pancreatic β cells. Transplantation of immature human islet-like cells to immunodeficient mice enables further maturation of islet cells. This is evidenced by human C-peptide secretion in glucose tolerance tests and by morphological and ultrastructural studies [2, 22, 23, 123]. It is important to understand what factors in the in vivo milieu are critical to functional maturation. These could be related to local signals provided by the in vivo niche at the transplant site. Recently, it has been shown that maturation occurs faster and more efficiently in female recipient mice, pointing to the potential role of estrogen receptor signaling in maturation [124]. Gene profiling studies suggest that the creation of mature β cells, in which insulin secretion is tightly coupled to glucose concentrations, requires the coordinated upregulation of certain genes and repression of others [125]. A recent report showed that ligand-dependent transcription factor estrogen-related receptor-γ (ERRγ) is a driver of the oxidative metabolic gene network in mature β cells and that its postnatal induction orchestrates the metabolic maturation of β cells [123]. This report also indicated that β cell-specific ERRγ-deficient mice are glucose intolerant and fail to appropriately secrete insulin in response to glucose challenge. ERRγ expression during postnatal β cell maturation drives a transcriptional program that promotes the mitochondrial oxidative metabolism necessary for GSIS. As such, promoting ERRγ expression and activity during the late stage of hiPSC differentiation in vitro results in glucose-responsive β cells that are capable of restoring blood glucose in type 1 diabetic mice. Thus, future studies to improve functional maturation of insulin-expressing cells should focus on signaling pathways that regulate the maturation of pancreatic β cells. (6) Low survival rate and immunogenicity: Because of the lack of a suitable transplantation technique, the recovery of well-demarcated grafts after transplantation into ectopic sites in experimental animals and examination of glucose responsiveness of hESC/iPSC-derived pancreatic cells in vivo are still difficult. This could be due to immune rejection in the host animals. At present, two different transplantation strategies are applied: one is direct implantation of hESC/iPSC-derived pancreatic cells into ectopic sites, and other one is implantation of a device containing induced pancreatic cells (Fig. 4). In the first method, pretreatment to induce angiogenesis at implantation sites is used to promote engraftment and long-term survival of the implanted cells. One recent study showed that a nylon catheter embedded into the subcutaneous tissues of host mice for 1 month before cell implantation generated a vascularized space [126]. The formation of vascular networks at the implantation site and the implantation of pancreatic cells after inflammatory reactions have diminished develope a less intolerant environment for the implanted cells. In the second method, pancreatic cells are encapsulated in a device made of biocompatible material that includes semipermeable membranes. Oxygen and nutrients can pass through the membranes to promote cell survival, differentiation, and maturation, whereas immune molecules and cells cannot. Several studies have shown that, when implanted subcutaneously into host mice, hESC/iPSC-derived pancreatic cells encapsulated by these semipermeable membrane devices can further differentiate into mature insulin-secreting cells and survive from host immune responses, primarily by T cells [20, 21, 25, 35]. In addition, because of vasculogenesis around these devices, the differentiated β cells can secrete insulin in response to changes in glucose concentrations. A recent study also demonstrated implantation of hESC-derived β-like cells encapsulated with an alginate derivative. This device mitigates foreign body responses and implants fibrosis, and induces glycemic correction without immunosuppression in immune-competent mice [127]. These device-based implantation methods can reduce or eliminate the need for immunosuppressive agents. Furthermore, these methods may have the advantage of allowing removal of the implanted cells with the device when adverse events such as tumorigenesis or dysfunction occur. However, it is presently unclear whether protection from soluble antibodies directed against differentiated β cells will be a significant problem [128]. Although insulin-expressing cell maturation can occur with or without the implanted cell/device combination, the survival rate of the implanted cells in hosts is still relatively low. Thus, a suitable transplantation technique, along with an effective combination of inducers, is required to overcome this transplantation challenge. Despite several limitations, phase 1/2 clinical trials have already started for the treatment of type 1 diabetes patients using a semipermeable membrane capsule device that carries hESC-derived pancreatic progenitors co-expressing PDX1 and NKX6.1 [36]. This trial has attracted attention worldwide, as it represents an important first step for the development of new stem cell therapies for diabetes. To date, no clinical trials with hiPSC-derived pancreatic cells have been carried out. However, the potential advantages of hiPSC over hESC may make such therapies available in the future. (7) Islet β cell heterogeneity: β cells in human pancreatic islets have been thought to be a homogenous cell population. Despite this prevailing paradigm, there have also been reports of β cell heterogeneity in human islets [129, 130]. Recently, a study by Dorrell et al. has identified four antigenically distinct subtypes of human β cells, which are distinguished by differential expression of ST8SIA1 and CD9 [131]. These β cell subpopulations are always present in normal adult islets and have diverse gene expression profiles and distinct basal and glucose-stimulated insulin secretion. Dissimilar basal and glucose-stimulated insulin secretion characteristics indicate that the β cell subtypes are functionally distinct. Dorrell et al. in their study isolated live pancreatic β cells from human islet samples by FACS and co-labeled them with antibodies recognizing ST8SIA1 and CD9 and finally identified four antigenically distinct β cell subpopulations. They also accessed the expression of ST8SIA1 and CD9 in sections of human pancreas and further confirmed the existence of four distinct β cell subpopulations in human islets. Transcriptome analyses by RNA sequencing of the β cell subsets have shown that most of the differentially expressed genes are of unknown function in β cells, but some have been clearly associated with insulin secretion or are known to be dysregulated in type 2 diabetes mellitus. Dorrell et al. have also observed that the frequencies of β cell subtypes are altered in the majority of individuals with type 2 diabetes. Thus, the β cell subpopulations may have relevance to diabetes and this issue needs to be addressed by extensive research. Almost all previous studies have attempted to generate mature β cells from human ESCs/iPSCs. Recently, researchers are mainly focusing on efficient techniques to enhance the yield of mature β cells and their successful survival post-transplantation to retain normoglycemia in diabetes. However, β cell heterogeneity is an emerging issue. The distinct properties of human β cell subtypes found in human islets likely have an important impact on metabolic regulation and human disease processes. Thus, future studies should focus not only on the generation of mature β cells but also on the formation of subpopulations of β cells from human ESCs/iPSCs and reveal their functional properties to potentially apply in diabetes.

## Conclusions

The ability to obtain a large number of mature insulin-producing cells from hESC/iPSC could provide unlimited supplies of surrogate β cells to replace damaged cells in patients with diabetes. Insulin-expressing pancreatic cells generated from hESC/iPSC to date appear similar to neonatal cells. Generating mature insulin-expressing cells with the same GSIS capability as endogenous β cells and their survival following transplantation into ectopic sites in experimental host animals are challenges for future research. The processes of β cell differentiation and islet organoid formation are controlled by a complex network that depends on transcriptional regulation of genes involved in pancreas development, a certain type and number of differentiation factors, and specific types and conditions of stem cell culture. Although many factors are important for the successful generation and transplantation of insulin-secreting β cells or islet-like organoids, a suitable combination of factors and conditions stands out as one of the most critical factors. Thus, more work is needed to increase maturity and post-transplant survival of hESC/iPSC-derived insulin-producing cells or islet-like organoids those resemble endogenous pancreatic β cells or islets, respectively. To achieve this goal, chemical screening of various regulatory factors and small molecules in late stages of differentiation in vitro may be a good option. A list of standard criteria for designing and performing future preclinical studies for hPSC-derived β cells/islet organoids in vitro and in vivo is given in Table 2. Although a number of considerations currently limit the use of hESC/iPSC-derived cells in cell replacement therapies, priority should be given to the issues discussed above.

**Table 2:** Standard criteria for designing and performing future preclinical studies in vitro and in vivo

Preclinical tool	Pros	Cons (challenges)	Possible improvements
	- NOD mouse is ideal for studying type 1 diabetes and the characterization of the immunopathology of the disease. - BioBreeding rat is a suitable model for understanding the genetics of type 1 diabetes [132] and studying of neuropathy-associated diabetes [133]. - Diabetes induction in the murine model is possible via the exposure to certain chemicals, namely streptozotocin and alloxan, and thus offers a useful tool for testing the potentials of therapeutic agents or transplanted cells to reduce glucose level.	- Some disease susceptibility loci in NOD mouse have no marked impact in human disease. - Various drugs and antibody therapy showed an excellent effect in NOD mice but no effect in the clinical trials [134]. - Induction of diabetes in NOD mice is correlated with microbial infections. - Induction of diabetes using chemicals in the animal model could show toxic effects to the other organs, such as the kidney, liver, brain, intestine, and reproductive organs.	- Applying humanized mouse model having the components of the human immune system. - Taking into account the gender-dependent diabetes pathogenicity in animal models. - Setting up new animal models that recapitulate diabetes pathogenesis in human. - Maintaining NOD mice under specific pathogen-free environment during diabetes experimentation. - Considering toxic actions in animal models during the chemical induction of diabetes in vivo. Previous reports showed the occurrence of lymphopenia and high production of T regulatory cells [135]. - For studying type 2 diabetes, the occurrence and the cause of obesity should be considered. - Studying diabetes complications (neuropathy) need to avoid selecting neuropathy-resistant mouse such as C57BL/6 strain [136].
Stem cell quality	- PSCs could obviate the hurdles of islet application such as lack of donors and weak secretion of insulin post-implantation. - Application of PSCs allows the understanding of patient-specific disease pathogenicity and also the development of potential therapeutics.	- Generation of iPSCs using integrative or viral-based methods hinders their clinical application in diabetes therapy. - PSC cultures using undefined or xenogeneic conditions produce cells having unusual characteristics and poor phenotypes, and thus cannot be applied in the clinic.	- Using non-integrative and safe methods for the generation of iPSCs. - Developing accurate assays for evaluating the quality of iPSCs, such as karyotyping, analysis of the pluripotency markers, and the differentiation capacity. - Developing efficient methods for heterogeneity and teratoma assays. - Microbiological assays for the detection of cell contamination, such as mycoplasma test. - Using defined and xeno-free culture conditions.
Organoid/spheroid culture	- Organoid/spheroid culture allows a detailed understanding of diabetes pathogenicity, molecular mechanisms, and disease model and provides a useful tool for drug screening. - For organoid culture, Matrigel, collagen-Matrigel, or hydrogels are mainly used as a platform.	- Application of animal-derived ECM such as Matrigel hampers the further application of generated organoids in the clinic. - Organoid culture is costly and laborious for the large-scale production.	- Designing suitable safe xenogeneic free scaffolds (physical cues) with growth factors (biochemical cues) for the generation of stem cell niche. - Discovering a cost-effective agents and protocols for efficient organoid culture at the large scale. - Developing efficient assays for the evaluation of the generated organoids/spheroids prior to their application for disease modeling or drug screening.
Differentiation methods	Various differentiation protocols are developed for the generation of insulin-producing β-like cells from PSCs in either monolayer or 3D culture using a cocktail of various chemicals, growth factors, inhibitors, and cytokines in order to emulate the in vivo system.	- Differentiation protocols depend on agents of high costs. - Many of the developed protocols are not reproducible. - The molecular mechanisms of most of the chemicals used in each step of the differentiation method remain unrevealed.	- Characterizing the reproducibility of the current β cell differentiation protocols from PSCs. - Setting up highly efficient protocols for the generation of mature β cells and their transplantation. - Culture conditions such as culture media, cell density, ECM, cell-cell, and cell-ECM interactions have an impact on PSC differentiation [68, 137–139] and thus should be optimized. - Characterizing the molecular mechanisms of the factors used in the current differentiation protocols.
Transplantation devices	- Encapsulation devices used for cell transplantation, such as semipermeable capsule or membrane, possess various functions [140]: ▪ Avoiding the undesirable host immune reactions against the transplanted cells. ▪ Protecting the patient from tumorigenic action of stem cells. ▪ Avoiding the loss of viability of the transplanted cells. ▪ Maintaining stable insulin secretion	- The encapsulation devices need the application of immune modulating agents. - Encapsulation devices may provoke the patient’s immune system and ultimately lead to cell death.	- Applying suitable agents with immune modulating functions, summarized previously [140], which protect the transplanted stem cells from rejection. - Designing an efficient encapsulation device with the following features: ▪ Allowing enough blood supply to the encapsulated cells. ▪ Having biocompatibility. ▪ Avoiding the stimulation of host immune reactions. ▪ Permitting the efficient transfer of the secreted insulin to the circulation.

## Abbreviations

BMP: Bone morphogenetic protein
DE: Definitive endoderm
EC: Hormone-expressing endocrine cells
ECCs: Endocrine cell clusters
ECM: Extracellular matrix
EGF: Epidermal growth factor
EP: Endocrine progenitor
ERRγ: Estrogen-related receptor-γ
ESC: Embryonic stem cells
FGF: Fibroblast growth factors
GCG+: Glucagon-positive cells
GHRL+: Ghrelin-positive cells
GLP-1: Glucagon-like peptide-1
GSIS: Glucose-stimulated insulin secretion
HGF: Hepatocyte growth factor
HUVEC: Human umbilical vein endothelial cell
INS+: Insulin-positive cells
iPSC: Induced pluripotent stem cells
KGF: Keratinocyte growth factor
MEFs: Mouse embryonic fibroblasts
MSCs: Human mesenchymal stem cells
ND: Non-diabetic
NKX6.1: NK6 homeobox 1
NOD: Non-obese diabetes
PDX1: Pancreas/duodenum homeobox 1
PG: Primitive gut tube
PI3K: Phosphatidyl inositol-3-kinase
PP: Pancreatic progenitor
PPY+: Pancreatic polypeptide-positive cells
PSC: Pluripotent stem cell
SCID: Severe combined immunodeficiency
SST+: Somatostatin-positive cells
STZ: Streptozotocin
T1D: Type 1 diabetes
TGF-β: Transforming growth factor-β
Wnt: Wnt signaling
